# Retrieving and Identifying Remnants of Artefacts on Local Devices Using Sync.com Cloud

**DOI:** 10.3390/s25010106

**Published:** 2024-12-27

**Authors:** Abdulghani Ali Ahmed, Khalid Farhan, Mohd Izuan Hafez Ninggal, Ghadir Alselwi

**Affiliations:** 1 School of Computer Science and Informatics, Faculty of Computing, Engineering and Media, De Montfort University, Leicester LE1 9BH, UK; 2 School of Computer Science and Engineering, University of New South Wales, Sydney, NSW 2052, Australia; k.farhan@unsw.edu.au (K.F.);; 3 Faculty of Computer Science & Information Technology, Universiti Putra Malaysia, Serdang, Selangor 43400, Malaysia; mohdizuan@upm.edu.my

**Keywords:** cloud forensics, cloud company services, web browser, Sync.com cloud, digital forensic, cyber investigation, cloud computing

## Abstract

Most current research in cloud forensics is focused on tackling the challenges encountered by forensic investigators in identifying and recovering artifacts from cloud devices. These challenges arise from the diverse array of cloud service providers as each has its distinct rules, guidelines, and requirements. This research proposes an investigation technique for identifying and locating data remnants in two main stages: artefact collection and evidence identification. In the artefacts collection stage, the proposed technique determines the location of the artefacts in cloud storage and collects them for further investigation in the next stage. In the evidence identification stage, the collected artefacts are investigated to identify the evidence relevant to the cybercrime currently being investigated. These two stages perform an integrated process for mitigating the difficulty of locating the artefacts and reducing the time of identifying the relevant evidence. The proposed technique is implemented and tested by applying a forensics investigation algorithm on Sync.com cloud storage using the Microsoft Windows 10 operating system.

## 1. Introduction

Cloud computing has gained significant recognition and popularity as technology has advanced, providing a wide range of computing services to numerous businesses and consumers. Organisations frequently utilise cloud computing resources to replace large, on-premises systems such as servers and data centers, ensuring high accessibility to data and reliable service availability for customers. This technology allows users to upload data to web servers, enabling instant access and the ability to share information with others at any time. According to National Institute of Standards and Technology (NIST), cloud computing represents a model for offering ubiquitous, convenient, on-demand network access to a shared pool of configurable computing resources (e.g., networks, servers, storage, applications, and services) that can be rapidly provisioned and released with minimal management effort or service provider interaction [1,2].

Small and medium-sized businesses profit from cloud computing as they can access the required computing services without having to spend a significant amount of money. Simultaneously, the great scalability of infrastructure resources gives businesses and IT organisations additional opportunities to try out high-end services with no upfront costs, particularly with a pay-as-you-go model that enables low-cost, on-demand computing. Cloud technology, on the other hand, creates a challenge for forensic investigators because data may be uploaded or shared from one computer and opened on another without leaving a lot of traceable evidence. Consequently, cloud storage services such as Dropbox^TM^, Microsoft^®^ OneDrive^®^, Google Drive^TM^, Flickr, iCloud, pCloud, and Sync.com worth further investigation [3].

Despite the advancements in cloud storage and the benefits it provides to all of us, we cannot ignore the fact that it is vulnerable to cybercrimes and provides an opportunity for cybercriminals to use it as a suitable medium for storing or transmitting their malicious content. One example is criminals using cloud storage for storing, transferring and launching botnet assaults. Given cybercrimes involving cloud storage continue to increase, the importance of cloud forensics also grows. According to the NIST, cloud forensics is the application of digital forensics techniques to collect, preserve, analyse, and interpret digital evidence from cloud systems. It involves investigating cloud environments to uncover evidence related to security incidents, data breaches, or other cybercrimes that may have occurred within cloud services or infrastructure. while ensuring data integrity and a strict chain of custody [1].

So far, digital forensics has dealt with the growing legal challenges that law enforcement will confront when attempting to collect or retrieve data from the cloud. Many businesses will then overlook the legal ramifications of cloud computing. According to Network World [4,5], Any organisation that plans to use cloud-based services should consider the following question: How can cloud providers supply us with digital forensics material in the case of a legal issue, civil or criminal incident, cyber-attack, or data breach? As a result, clients of public cloud services sought their cloud provider’s help in investigating and retrieving digital evidence stored on clouds. The cloud provider should conduct each search on behalf of the requester to obtain the digital evidence. The forensic investigators are then required to ensure that the evidence gathered is genuine, trustworthy, complete, convincing, and admissible [6,7]. Furthermore, the difficulties of accessing physical hardware to identify files and evidence make the evidence acquisition procedure more complicated, since all data are strewn around the globe on various distributed computers and data centres that may or may not belong to the same businesses. In addition, to collect cloud data without breaching individuals’ privacy and security, law enforcement authorities employ the time-consuming search warrant process in order to instantly collect pertinent data.

Cloud computing systems presents challenges for a computer forensic analyst in acquiring and analysing digital evidence up to the standards expected for traditional server-based systems. This difficulty arises from the complexity of determining which data were stored or processed by specific software on particular computing devices [8]. To ensure that all collected digital evidence is appropriate and meets the requirements for court hearings while also being understandable by jurisdiction personnel without IT backgrounds, a proactive methodology for conducting digital forensic investigations is crucial. This methodology should also be adaptable to accommodate future providers offering similar services [9,10]. Furthermore, it is essential for forensic investigators to understand the nature and locations of data remnants that cloud users may inadvertently leave behind on the devices they use to access their data.

In this study, we aim to conduct a thorough and efficient investigation that focuses solely on cloud clients rather than cloud servers. We then developed a technique for identifying the location of data remnants to assist in collecting digital artefacts from cloud client browsers. Additionally, our technique aims to analyse the collected artefacts and help to identify digital evidence relevant to cybercrimes. Sync.com cloud storage is used as a case study and serves as the foundation for the experiment in our research. The main scope of this study is defined by its functionality, users, data, and system platform. We employ a lab-based methodology designed to assist in locating digital evidence fragments during cybercrime investigations within cloud environments, especially when sensitive data are involved. The potential beneficiaries of this research include cloud forensics investigators and government agencies responsible for managing sensitive information. Data are collected by running Sync.com cloud services within virtual machines set up on a Microsoft Windows 10 platform. The experiments are conducted using the Microsoft Windows 10 operating system, employing various monitoring and forensic tools to facilitate the testing process.

The contribution of this study is to reduce the time needed to locate, retrieve and investigate artefacts. That means reducing the time required to retrieve artefacts from the cloud after a crime has occurred. With the approach and strategy that we will present in this study, the time spent by cloud investigators today on collecting associated data, information and keys for evidence will be reduced, facilitating the forensic investigation process and making it faster and more efficient. The contributions also include enhancing the accuracy of retrieving sufficient evidence relevant to cybercrime. Furthermore, this study contributes to enhancing the capability to retrieve as much evidence as possible, assisting forensic investigators in collecting abundant evidence for their investigations and court proceedings. Therefore, the findings of this study will assist cloud investigators in pinpointing the exact and precise location of the data required as evidence for their cases.

The rest of this paper is organised as follows: Section 2 provides background information on cloud forensics and explains the primary investigation process models in the field of digital forensics. In Section 3, the literature review is discussed, focusing on the analysis of previous studies regarding data collection and investigation in the cloud environment. This section also critically evaluates the strengths and weaknesses of existing works in comparison to our proposed technique. Section 4 outlines the methodology proposed in this study. Section 5 presents and discusses the results and findings obtained. A comparative analysis between our findings and those reported in related works is provided in Section 6. Section 7 concludes this study and highlights potential areas for future research.

## 2. Background and Investigation Process Models

We discussed the introduction of our study in Section 1, which covers the problem description, aim, and contributions. This subsection provides background information, explaining the existing digital evidence collection approach in cloud forensics, and focusing on describing the primary process models used for forensic investigations. Cloud computing, as we know, is a type of on-demand computing that provides computers and other internet-connected devices with pooled processing resources and data. The assumption is that, according to Gartner, Inc. (Stamford, CT, USA), global end-user spending on public cloud services is projected to increase by 18.4% in 2021, reaching a total of $304.9 billion, up from $257.5 billion in 2020 [11]. Forensics, on the other hand, is the use of scientific tests or methodologies for the detection of crime. As a result, cloud forensics may be characterised as a multidisciplinary approach to criminal investigation that incorporates cloud computing and digital forensics.

### 2.1. Types of Clouds Service Providers

Several cloud storage services cater to diverse user needs. Dropbox supports multiple operating systems and offers 2 GB of free storage, expandable to 16 GB through referrals, featuring file versioning and AES 256-bit encryption via Amazon S3. Google Drive integrates with Google accounts, providing 15 GB of free storage, real-time collaboration, and a 5 TB file size limit, secured with 128-bit AES encryption and two-step verification. Microsoft OneDrive offers 15 GB of free storage, with unlimited options through Office 365, encrypting data during transit, and providing two-step verification. pCloud gives 10 GB of free storage, which can increase to 20 GB through referrals, allows unlimited file size uploads, and features a Crypto Folder with 256-bit AES encryption for enhanced security [3]. Lastly, Sync.com cloud drive provides 5 GB of free storage with zero-knowledge encryption, enabling multiple users to collaborate, recover deleted files, and archive data through Sync Vault, ensuring the easy management and backup of important files.

Each of these services has unique strengths tailored to specific use cases, from casual users needing basic storage to businesses requiring advanced collaboration tools. Users should consider factors such as ease of integration with existing workflows, scalability, and specific security needs when choosing a provider. Additionally, the ability to recover deleted files and the extent of collaboration features can significantly impact productivity, making it essential to assess how well each option aligns with personal or organisational requirements.

From a digital forensics’ perspective, understanding how cloud services handle data remnants is crucial. Even after deletion, fragments of files may persist in the cloud, potentially exposing sensitive information. Different platforms have varying policies on data retention and deletion, which can complicate forensic investigations. Forensic experts need to consider the methods used for data overwriting and the service’s approach to data encryption, as these factors can affect the recoverability of deleted files. Additionally, logging practices and user activity records can provide valuable insights during investigations, highlighting the importance of choosing a service that maintains comprehensive audit trails for accountability and transparency. Table 1 provides a summary of each cloud’s storage services.

### 2.2. Investigation of Computer-Based Crime Guideline

With the rise of cloud computing and the increase in cybercrime, forensic investigators have a variety of options for acquiring and preparing legal digital evidence to present in court. Existing evidence acquisition methods are explored in detail to meet the objectives of this study. In general, digital forensic practitioners and examiners adhere to approved standards, norms, and procedures. Identifying, conserving, analysing, and presenting evidence in a legally admissible way are the four main procedures of forensic computing, according to [12] procedures. The details of each procedure are described as below:

- Identification: This procedure entails determining the sort of digital evidence discovered, its storage components, and the evidence’s location, as well as the method that aids in data recovery. The type of data kept and the format in which it is stored should also be researched in advance to select the best tools for extracting it.
- Preservation: When it comes to data preservation, it is critical to make sure that the inspection of electronically stored data is carried out in the least intrusive way possible. This is to assure the data extraction’s integrity and evidentiary relevance.
- Extraction: Extraction processing and the interpretation of digital data from electronic devices are all part of the analysis process. The content stored within the evidence, for example, is extracted and processed in a comprehensible manner.
- Presentation: This phase entails the actual presentation of evidence gathered in a courtroom.

The United States Department of Justice (DoJ) established a four-phase process model in its electronic crime scene investigation handbook for law enforcement in 2001 [13]. However, in the second edition of the guidance [14], this model was broadened to encompass the processes of preparation, preservation, documentation, collection, examination, analysis, and reporting. The details of each process are explained as below:

- Preparation: This procedure includes understanding the many types of electronic devices and computer systems often encountered, as well as prospective evidence sources, handheld devices, investigation tools, and equipment for collecting, packaging, and transporting electronic evidence.
- Preservation: This procedure entails securing and evaluating the crime scene, as well as guaranteeing the safety of those in the vicinity of the crime scene while also protecting the integrity of all evidence gathered there.
- Documentation: This procedure includes documenting the situation in order to compile investigation reports and electronic evidence.
- Collection: This procedure includes gathering digital evidence from the previously confiscated computer and storage devices. To guarantee the integrity of the evidence being collected, none of the evidence should be tampered with, and the hash value of the files before and after copying should be the same.
- Examination: This method includes displaying the evidence gathered and explaining its origin and relevance in relation to the crimes.
- Analysis: This procedure entails assessing the examination’s outcome for its importance and probative value in the case.
- Reporting: “An examination is concluded with the preparation of a report that summarises the examination technique as well as the important information discovered”.

We used the technique suggested by [12,13,14] in this study, which covers the phases of identifying, preserving, analysing, and presenting evidence. The primary goal of this study is to use the newly formed cloud storage service ’Sync.com’ to perform quantitative and qualitative research to identify whether network traffic contains information useful for forensic investigation and whether that information is still stored in memory. The goal of this research is to investigate what data are left on a computer hard disc after a Sync.com user has utilised client software and where the data remnants are located within the Microsoft Windows 10 operating system. It is also to identify what data remain on the PC hard drive after a Sync.com user has accessed cloud storage via a web browser. By finishing this study, we will attain a greater grasp and awareness of the remaining artefacts and their locations, which will aid cloud forensics investigators in the data gathering and investigation of digital evidence in the Sync.com cloud.

## 3. Literature Review and Related Works

Within this section, we review the current state of the art in our study to gain a deeper understanding of cloud forensics. This section is divided into two subsections: a discussion of related work and a critical analysis that examines the advantages and limitations of the related works in comparison to our proposed technique.

### 3.1. Related Work

In this section, we reviewed papers and studies which are related to cloud forensics with focus on the most related studies that deals with collecting and investigation possible evidences left at the client devices. For each paper assessed, a short-term overview of the research findings, methods, limitations, and conclusions is provided, and any comparable conclusions or contradicting findings are examined further below.

To begin, a study conducted in [15] to find data traces on a Microsoft Windows 10 device by examining Amazon Cloud Drive’s cloud activities and identifying evidence that may have been left behind through the use of various Internet browsers. In this paper, the authors utilized Amazon Cloud Drive as a case study to gather evidence from criminal devices using the EnCase forensic tool. The primary focus involved analysing VMDK files that had been deleted using a wiping forensic tool like BleachBit, applied across various Internet browsers to enhance the volume of collected data, thereby aiding in improving the investigation process. The researchers explored Amazon Cloud Drive’s cloud activities and tried to find some clues that could have been left behind using various Internet browsers. The researchers only focused on the username, the cache files, and log activity, but they did not focus on files that had been uploaded, downloaded, synced, deleted, etc., to enhance the efficiency of the digital forensics and crime investigation.

In Ref. [16], the same authors conducted research on Google Drive’s cloud. The authors explored data remnants left on a Microsoft Windows machine from Google Drive’s cloud activities, aiming to improve the digital forensics and crime investigations. They used EnCase to analyse VMDK files and recover deleted data but found it to be costly and slower, particularly when dealing with large files. On the other hand, FTK (Forensic Toolkit, version 7.5.1) provides a faster alternative in terms of its parallel processing feature, which helps speed up the analysis of large datasets. FTK is also simpler to use, offering tools such as a dynamic evidence search, hash-based indexing, and more effective recovery of deleted files, making it an efficient choice for investigators handling vast amounts of data.

In addition to its speed and ease of use, FTK stands out in other areas, such as cloud and browser data analysis. It comes with built-in tools for email analysis and reconstructing Internet history, which are useful for examining evidence left by Google Drive and browser activity. FTK’s superior handling of cloud-based evidence, including its ability to extract metadata from cloud files, provides an edge in modern forensic investigations. Although the authors focused on browser data, they noted that FTK’s strength in recovering deleted files could have been better utilised, especially given the increasing challenges posed by newer data deletion technologies.

On another note, we discovered that [17] had previously undertaken research to see if Dropbox data could be uploaded, saved, and then acquired. The researcher used Dropbox on Ubuntu 16.04 LTS to create a Dropbox account on the web, install it on a Linux machine, and sync it with the operating system, resulting in the creation of some files in the Linux home and uploaded files from another operating system via Dropbox’s website being synced and viewable in Linux as well, after which they deleted their files. They then used a recovery tool called Testdisk for the recovery of data. They also utilised the terminal to generate a File CreatedInLinuxDropbox.txt file at the same location using text files, such as .pdf and FileCreatedInLinux.txt, that had been uploaded to Dropbox. Following that, the files were erased and examined. Using the rm command in the terminal, files were removed while keeping track of their properties. When using the rm command, extra caution is essential.

In this research, the researchers also used the PhotoRec Linux tool for the recovery of data. Photorec is free, and it is also an open-source program. It stores different file systems in blocks, such as NTFS and ext3/ext4. PhotoRec is a program that recovers data from files that have been lost or destroyed on a variety of file systems. At a certain location, these blocks are stored in sectors. Every operating system aids in the continual storage of these data for improved performance. This method involves a lot of reading and writing operations. The researchers also tested the overwriting of a memory region with other evidence and discovered that the old data could be restored even if the file was deleted. PhotoRec calculates the block size during this step. After determining that the file is corrupted, the utility Photorec begins reading sectors and saves the first ten files. These files assist in determining the block size. After then, data can be fetched from that location block by block. These can be compared to the database to ensure that they are recovered in the correct format. Because of their study, the researchers discovered that copying huge files to the given place only takes seconds. Large data files on a hard disc may take minutes to load. Files, such as .mp3, .doc, .pdf, .mp4, .exe, and others, can be recovered. They also discovered that when they created a doc file on the Dropbox website, it instantly synced with Linux. This doc file was also viewable and editable in Libre Office. After testing, the accuracy of total file restoration in Testdisk was 97%.

The researcher also discovered that the Dropbox app stores a few devices that are used to access or sync with the account. Each timestamp of its Dropbox activity, such as installing, uploading, downloading, and uninstalling specific applications and data, may also be found. In addition, data recovered by the tool’s proper location can assist in identifying possible objects in cloud storage applications for forensic investigators.

Research presented in [18] conducted a study to uncover evidence data on a client computer. This study aimed to offer forensic practitioners insights into the types of evidence present in machines using Amazon Cloud Drive and Microsoft OneDrive. The purpose of this research was to look at the capability and scientific correctness of these forensic toolkits in obtaining forensic data from cloud computing environments over the Internet. The authors used data for forensics professionals to classify the evidence data on a client computer to provide information about the type of evidence that resides in computers. Data timestamps, file hashes, client program log files, memory captures, connection files, and other facts discovered during this analysis were all available to various cloud service providers.

They utilised a browser and a service provider’s application to undertake forensic evidence gathering on a Microsoft Windows 7 system, utilizing two common public cloud service providers (Microsoft OneDrive and Amazon Cloud Drive). File timestamps, link files, file hashes, memory grabs, client software log files, and other evidence were also available to multiple cloud service providers, according to the researcher. At the conclusion of this study, the researchers found that investigators are able to collect user account information. The collected information will aid investigators in predicting the location of user data and taking appropriate action to gather, evaluate, and protect this evidence in a timely manner. By executing numerous operations, such as hash comparison, registry analysis, RAM analysis, keyword searches, and network packet traffic analysis, the authors discovered that the investigator could identify cloud storage account usage details. The authors also stated that by collecting and analysing a computer’s RAM dump and network packet data, the investigator would be able to obtain the login and password for the OneDrive account, as well as the username and password for the Mega account.

Furthermore, ref. [19] conducted research to analyse thoroughly both the Mega cloud drive and the IDrive client software, aiming to uncover the types of evidence left behind on a user’s computer by these cloud services. To carry out this research, the authors set up the Mega cloud drive and the IDrive account, uploaded and downloaded data, and then used forensic software to erase it all from the OS they were using.

Refs. [20,21,22] conducted research on pCloud and Dropbox on Microsoft Windows 10 and Microsoft Windows 7 OS with the goal of locating data evidence on client computers and presenting forensic practitioners with a clear image of the forms of evidence that may be found on machines that have the two cloud providers installed (pCloud Drive and Dropbox Drive). The authors evaluated two cloud service providers in this study, which explained the many procedures involved in obtaining evidence on a user account using a browser and then via a cloud software application on a Microsoft Windows 7 machine (IDrive and Mega cloud drive). However, this research established that objects on a user machine would provide investigators with a general understanding of the type of evidence residue in the user device. Therefore, registry data, program logs, file time and date values, and browser objects were among the main pieces of evidence discovered during this investigation on a user’s Microsoft Windows machine from these two cloud firms.

Refs. [23,24] highlighted that many researchers have tried to find and address the problems in cloud computing by proposing new approaches and techniques. The authors highlighted a few of these issues and explored several possible options for overcoming them. Log files, volatile data, creating forensic images, and data integrity were among the challenges addressed in their report. The researchers discussed some of the difficulties investigators encounter while investigating crimes committed utilising the cloud. They came up with potential solutions, such as gathering logs, volatile data, creating forensic images, and maintaining data integrity. They conducted collections, examinations, analyses, and reporting for logs, volatile data, forensic images, and data integrity for cloud analysis and testing using IaaS. The authors concluded that IaaS was the best model for investigators because clients have control over the system where all the evidence is stored.

In order to overcome the drawbacks of conventional digital forensic technologies, the researchers [25] proposed a novel framework for tracing leftover files through data remnant analysis. The technique finds and examines all data leftover in the system, methodically building a dataset depending on user input. The findings support digital forensic investigations on Microsoft Windows systems by demonstrating more efficacy and accuracy when compared to current techniques.

In the digital age, electronic evidence is crucial for investigations and legal proceedings, yet traditional forensic methods struggle with recovering deleted data and unallocated spaces. The authors of [26] introduced a novel framework that they claimed enhances the tracing of residual files through data remnant analysis, enabling the identification and examination of traces left by deleted or overwritten data. By creating a systematic dataset based on user actions, the framework reveals previously undetected file traces. They used Microsoft 365 as a case study to illustrate the framework’s superior efficacy and accuracy compared to existing methods, offering significant insights for digital forensic investigations on Microsoft Windows systems.

The authors of [27] presented Search and CompAre Reverse (SCAR), an approach inspired by bioinformatics and designed to identify partial patterns in files that individuals are prohibited from possessing or that have been deleted by disgruntled employees. They asserted that SCAR could recover sensitive information, as remnants may still exist in cloud storage. This method represents an initial empirical exploration within digital forensics domain.

Similarly, the authors in [28] examined data recovery in the digital era, emphasising various approaches to protect digital assets. They covered types of data loss, backup strategies, industry standards, best practices, challenges, legal and regulatory concerns, and technological advancements. The focus was on the significance of effective backup strategies and the evolving landscape of technology in safeguarding data, with the goal of fostering a more secure digital environment.

Most issues in digital forensics stem from the collection and pre-processing of evidence, particularly due to counter-analysis techniques and the challenges involved in retrieving data remnants from cloud storage systems [29].

### 3.2. Critical Analysis

As we can see from the examples above, each of the preceding studies had some limitations. These limitations could be tightened even more to produce more precise and desirable research papers in this field. According to some estimates, the cloud storage business is now congested, with more than 2200 companies active around the world. As a result, research into cybercrime evidence collection on cloud storage services need to not be restricted to well-known cloud storage providers, such as Amazon, Google Drive, Microsoft OneDrive, Dropbox, or Sync.com Cloud. It should be expanded to software with recently formed cloud storage services to assist forensic investigators in obtaining evidence of various clouds. This is because the locations of data remnants kept in the computer hard disc after a user has used various types of cloud storage services may fluctuate based on the cloud storage services, they use.

Thus, in this research, “Retrieving and Identifying Remnants of Artefacts on Local Devices Using Sync.com Cloud”, we focus on the so-called newly established cloud storage service, Sync.com. Sync.com Cloud is a relatively new service, having been live since 2011. This solution is managed by Sync.com Ltd., a Canadian company that aims to build a “better new model that makes the existing one outdated. This study looks at the data that remain on a computer hard drive after a Sync.com user has used client software, the location of data remnants within the Microsoft Windows 10 operating system’s memory (RAM), and the network traffic that can be captured after a Sync.com user has accessed cloud storage via a web browser. All of this information is critical in assisting forensic investigators in obtaining evidence from criminals that use Sync.com cloud. The discrepancies in MD5 hash values that they acquire during their trials have a few limitations. MD5 has been employed in a wide range of cryptographic applications, and it is also often employed to check data integrity and the authenticity of electronic evidence challenged in court. To confirm that the original file is not altered in any manner, the MD5 hash value of the files must be the same before and after the data acquisition.

As a result, we use cryptographic hashes in this study to confirm that the files are identical in all circumstances. We carefully and methodically execute all testing and experimentation to guarantee that the original evidence information is not tampered with and that the data integrity to be presented in court is maintained. Finally, this research provides fresh insight into the process of identifying and gathering data that remain available on each personal computer hard drive, allowing forensic investigators to investigate each cybercrime and bring the perpetrators to justice.

## 4. Methodology

This study retrieves and identifies remnants of artefacts on local devices when accessed, uploaded, and downloaded through the Sync.com cloud drive, with a particular focus on how various browsers manage the remaining data. Unlike previous studies that have used general cloud storage models or only one browser, this paper examines Microsoft Internet Explorer, Mozilla Firefox, and Google Chrome to observe any browser-specific differences in how data remnants are handled using the cloud provider application (Sync.com). The goal is to determine whether different browsers leave behind unique traces, such as usernames, passwords, files, metadata, or other remnants from cloud interactions. The study also evaluates third-party tools, such as Eraser and CCleaner, to test their ability in removing traces left by the Sync.com cloud drive. By running these applications after cloud interactions, we aim to see how effective they are at erasing evidence, such as browsing history, file remnants, and login details.

Furthermore, this research analyses how data remnants accumulate after multiple cycles of accessing, uploading, downloading, deleting files, and/or installing applications. This will help determine if repeated interactions with cloud applications result in more persistent data over time, providing insights into how cloud storage handles data in the long term. The research follows a modified iterative waterfall model, chosen for its clear structure and defined timelines. This approach allows for the identification and resolution of issues, such as inconsistencies between browsers or missing evidence, throughout the process of data collection and analysis.

### 4.1. Requirement Definition

To begin with, the requirement definition stages are the initial phase of iterative waterfall models, and they involve brainstorming exercises to determine what type of research topic we should pursue. The steps in the requirement definition process involve identifying the problem statement, the aim, and the scope of the investigation, and gathering all relevant resources. The information obtained will mostly consist of acknowledged standards, regulations, and processes that digital forensic practitioners and examiners should follow to collect evidence in a forensically sound manner. We also look at methods for locating bits of data that were left on a Microsoft Windows 10 PC after using Sync.com cloud, such as login, password, software access or browser, files stored within the account, data remnants from the files stored, and the associated dates and hours. Every resource is significant and must be thoroughly examined for this research to proceed smoothly.

Several challenges and limitations are identified during the requirement definition stage, and a clear objective is proposed, which will serve as a guideline for us while conducting the research and testing trials. We were able to find three key objectives in doing this research, as indicated in the objective section. The resources offer details on how to achieve the results as well as the exact hardware and software required to assist with the research experiments. These essential findings were primarily gathered through online publications, as well as online e-books and former students’ references, in addition to reference texts available at the UPM Library and UPM Ezproxy.

### 4.2. Resource Analysis

Following the collection of all resources, we analyse each piece of information to see if it is appropriate and compatible with the research requirements, ensuring that it is valid for the construction of the application in this study. Throughout this stage, any errors discovered during the previous phases can be reviewed to remedy the errors that have been made. After that, all the information is finalised in order to choose the suitable method to be used in this research. As previously discussed, there are widely accepted standards, norms, and methods that digital forensic practitioners and examiners should adhere to in order to collect evidence in a consistent and forensically sound manner. As a result, we follow the guidelines as set out in [12,13,14] in this study. These principles necessitate minimal treatment of original evidence, accounting for all changes, adhering to evidence rules, and not exceeding knowledge, allowing us to define and specify four steps in the forensics investigation process. The process of identifying digital evidence, the process of preserving digital evidence, and the process of analysation are all examples of these specific steps.

As all know, one of the most common problems encountered during the evidence analysis process is locating potential data. This is valuable as a guide for forensic investigator examiners regarding what data to look for when cloud storage such as Sync.com is suspected. To gather all the essential resources, we compiled a list of relevant publications and past research papers and conducted a critical study of them. This study is critical in determining the methods they utilised to conduct their research, as well as their objectives and study limits, so that we might avoid the constraints we discovered. The data are hidden using anti-forensics methods to hide signs of cloud storage usage, and whether the data remain will be effectively identified as part of this research. Therefore, the resources discovered should include which cloud storage is used, the login and password entered, and any files associated with cloud account usage that may be important to the investigation methods and serve as a guideline as we conduct this study. In addition, we gather data on the many types of cloud service apps to explain their various advantages and disadvantages in general.

#### 4.2.1. Analysing Software and Hardware Requirements

The analysis also includes research and analysis of software, hardware, and some essential programming languages that must be understood before moving on to the design step. Because each platform has its own system architecture and kernel functions, the platform analysis procedure is critical before planning the overall system. Table 2 and Table 3 show the requirements of software and hardware used to conduct this research’s experiment respectively.

#### 4.2.2. Analyse the Scope of the Study and the Number of VMs Required

The objective of this research is to find out what data were left on a Microsoft Windows 10 PC after utilising Sync.com cloud, such as usernames, passwords, browser or program access, files stored within the account, data remnants from the files stored, and dates and times connected with the files stored. To accomplish this, three control base files are utilised to compare the succeeding process to ascertain the modifications made to each of the processes in progress. There is a requirement to perform known interactions with Sync.com cloud, such as the process of creating and accessing accounts using a variety of browsers and client software. We deployed 21 virtual computers to acquire the information and data needed to answer the research question utilising Sync.com cloud as a case study. We created several distinct scenarios, including using Microsoft Edge (ME), Mozilla Firefox (FF), and Google Chrome (GC), to access Sync.com using different browsers in order to compare the results.

The basic systems were built with 20 GB of RAM and hard drive space to restrict the amount of storage space necessary for the number of virtual devices and forensic images produced during the tests. We also wanted to cut down on the time it took to analyse the data generated by the tests. Third, it was thought that if relevant data could be found on smaller systems, there was a greater likelihood that the data would be found on the bigger system. We utilised a SysInternals Process Monitor to keep track of system changes in the virtual computer. However, when using the SysInternals Process Monitor, research has shown that forensic images and memory grabs are polluted with a considerable quantity of data in terms of both memory captures and hard drive pictures. As a result, we conducted this study without using the SysInternals Process Monitor. We were able to see changes to the registry files and file systems by comparing the base image files with succeeding image files to ascertain the changes made. Types of virtual machines needed for our experiment are described in Table 4.

### 4.3. System and Software Design

**Figure 1:**
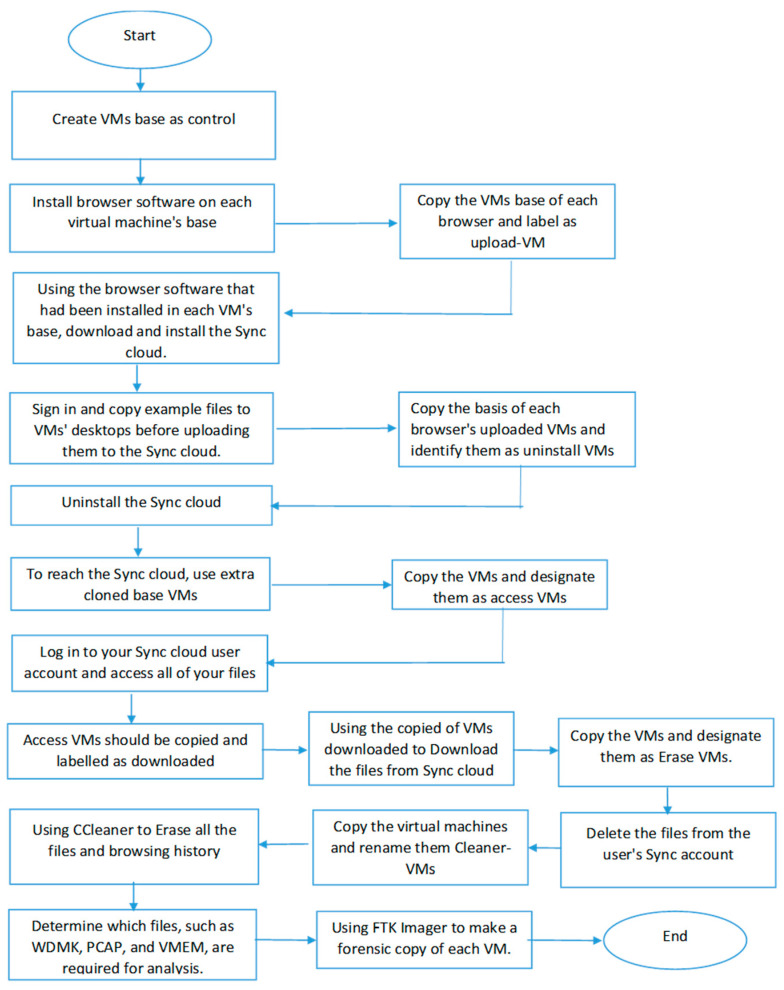
Flow chart of the experiment.

During this phase, all the data analysed are translated into a flowchart format. All the analysed requirements and information gathered throughout the requirement definition and analysis phase are applied in the system design phase, allowing us to apply it to our study. During the system design phase, we design the overall process flow carried out later during the implementation phase. This is also utilised to lead us through all the experiments we planned so that we can obtain our desired results within the time frame we set. It also gives an idea of how we performed our study experiment to save time in the future. Aside from that, the data we gathered allow us to standardise the specifications necessary for carrying out our study experiment technique. With this information, we construct a flow chart for how the experiment should be conducted during our trial, as well as how we tested the components later. The flowchart of the whole process is shown in Figure 1.

**Figure 2:**
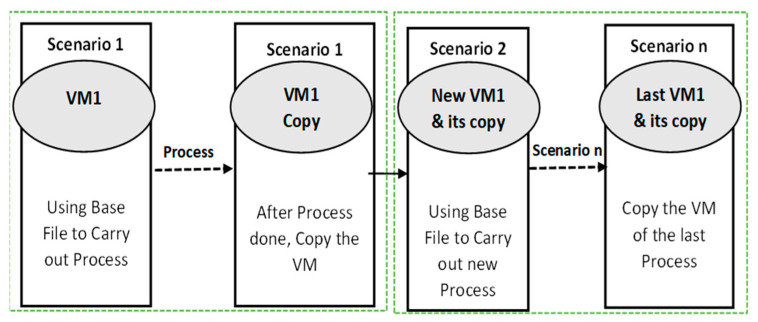
VM used during scenario execution.

In addition, the flowchart was utilised to direct the execution of planned experiment situations to accomplish the intended outcome within a set time. To prevent wasting time, this approach also offered an overview of how the experiment was carried out. Each procedure made use of fresh VMs that were cloned, removed, and reinstalled after testing to guarantee that each scenario was produced in a new VM environment and that accurate data were collected. In the newly generated VMs, the following situations were tested. Figure 2 depicts the use of virtual machines (VMs) during the scenarios.

### 4.4. Implementation and Testing Phase

The specifications gathered during the design phase are critical for the implementation of this research. The experiment is conducted with 21 virtual machines (VMs), using 6 computers, each assigned 3–4 VMs to optimise time and resources. Before starting the experiment, the system and software specifications are reviewed to ensure smooth execution. All the necessary hardware and software are set up in advance to avoid delays during testing.

#### 4.4.1. Experimental Setup

We improved our methodology by clearly defining the role of each Virtual Machine (VM) and incorporating more precise testing stages. Instead of vague labels, each VM was categorized based on its specific task in the experiment: Upload-VM, Download-VM, Uninstall-VM, CCleaner-VM, Erase-VM, and Delete-VM. This distinction helped to eliminate any confusion and made the function of each VM within the experiment straightforward. Moreover, we optimized resource allocation by assigning three to four VMs to each of the six computers used in testing, ensuring that the process runs efficiently without overloading any single machine. This configuration allows us to reduce time while maintaining consistent and reliable results. The assignment of specific VMs to each machine, with time constraints and resource management in mind, ensures that every VM serves its purpose effectively without unnecessary delays.

We also structured the testing phases within the experimental setup. After configuring the base VM, we introduced well-defined testing stages to verify the function of each VM and process before advancing to the next stage. These steps were crucial for maintaining the integrity of the experiment and ensuring that each process ran as intended. To further strengthen the methodology, we introduced scenarios simulating real-world challenges, such as data corruption, network latency, and network instability. These scenarios helped us evaluate how the system performed under less predictable conditions, which mirrored how these systems would behave in a real environment. This made our research more relevant and practical, addressing the complexities of cloud-based interactions.

Security and data redundancy were critical considerations throughout the experiment. To prevent unauthorised access or data manipulation during syncing between the VMs and Sync.com cloud, we employed encrypted communication protocols. This added layer of security ensured that sensitive data were protected throughout the process. Additionally, we incorporated redundancy testing to assess how the system handled multiple copies of data stored across different platforms. This ensured that no data were lost or corrupted, further enhancing the reliability and robustness of the cloud interactions being analysed.

Our methodology also addressed error handling and recovery processes. If the Sync.com cloud failed during file transfer, automatic retries were initiated and failures were logged for analysis. This system ensured that disruptions did not halt progress and that each step was recorded for later troubleshooting. Furthermore, we included a recovery procedure for VM crashes, which allowed us to restore the system to its last stable state without compromising the integrity of the experiment. This procedure ensured that even unexpected issues did not derail the research, providing a safeguard to maintain consistent results.

The collection of forensic data was significantly enhanced. Instead of simply capturing the VM state, we also analysed the network traffic during interactions with the Sync.com cloud. By using tools such as Wireshark, we captured relevant data and potential leaks that could indicate hidden traces left behind by cloud interactions. Additionally, FTK Imager was used to create detailed forensic images of the VMs, ensuring a comprehensive record of the experiment’s data. This two-pronged approach guaranteed that we identified all possible traces that may be left by the cloud service. We expanded residual data analysis to include system logs, memory dumps, and browser-related remnants, such as cookies, cache, and browsing history, to fully understand how cloud services interact with system data and to uncover any remaining evidence that could be vital for forensic investigations.

Finally, we introduced extensive data tracking and logging to monitor every file uploaded, downloaded, or erased during the experiments. Detailed logs captured timestamps, file information, and any remnants left behind after each process, ensuring thorough documentation for post-experiment analysis. These logs not only help in assessing the experiment’s progress but also provide insight into how different browsers handle residual data. For instance, we tracked browser-specific traces, including cookies, cache files, and browsing history, to understand how different browsers leave behind data after interacting with cloud services.

By incorporating these improvements, we made the experimental setup more rigorous and robust, ensuring that it could handle a wide range of real-world scenarios. This methodology provides a more comprehensive, accurate, and reliable way to assess cloud interactions, ultimately leading to deeper insights into data handling and forensic analysis in cloud environments.

#### 4.4.2. Integration and System Testing

Integration and system testing are the final steps in the process. This phase involves combining and testing the software modules together. We tested each function individually, as planned. The main goal here was to test the system in real time to confirm the accuracy of the results. This phase also helped to identify any flaws or limitations in the experiment, allowing us to adjust as needed.

Alpha testing was carried out, making any required changes to the software before beta testing. Commercial off-the-shelf software underwent alpha testing as part of the internal review process. Finally, the results of the performance tests and any leftover files were analysed to ensure that the experiment met the research objectives.

## 5. Experimental Results

The goal of this stage is to go through the implementation stage in depth, covering the steps involved and the sorts of tests that were conducted. The implementation stage consisted mostly of the processes for analysing the web browser Sync.com cloud account information, creating control images for each VM situation, and installing Sync.com client software. The client software uninstallation procedure, directory listing, prefetch, registry, network PCAP, browser, RAM, and link files were also examined and addressed. Finally, went over the results of the tests and the analysis that was carried out. The following subsection examines the files generated because of the scenarios that were run, as well as the files’ locations. Each of the forensic image files, network PCAP files, and memory dump data recovered from each scenario were copied from the hard discs in this study. FTK v7.1, HxD Version 1.7.7.1, Wireshark Network Analyzer 3.6.5, and other programs were used to view and analyse all the forensic photos copied. The analysis procedures were carried out to determine the content of the data remains that had been produced and left on the hard discs of the PCs. This data remnant helps forensic investigators in capturing images of the client’s activity, as well as other pertinent data.

### 5.1. Sync.com Cloud Account Information Using Web Browser

The username is shown in the upper right-hand corner of the browser when accessing www.sync.com on 3 February 2022, using different web browsers as specified. In addition, the number of devices synchronised with the Sync.com cloud account, including mobile phones and PCs, was displayed, as well as the amount of space utilised and accessible to the user. In addition, the information of the files and folders saved was posted on the Sync.com website homepage. This contained the file size and the folder’s latest modification date. There was also a file info icon in each file that allowed the user to access the file’s details.

We could have altered our account settings, as well as other options such as download links and connected account settings in the upper right-hand corner of the browser. We could have exposed or concealed system secret files, as well as reset account passwords, under account settings. There were also active tokens for the devices that are used to access the accounts, as well as some other associated information, such as the date the token was produced and the date it expired. In the download link settings, there was an option to show download links individually, as well as full usage metrics. Furthermore, Sync.com offers customers the option of backing up their folder and file contents to other accounts such as OneDrive, Facebook, Instagram, Dropbox, and Picasa Web Albums.

Using a web browser to access our Sync.com account also allowed us to examine our deleted files under the trash option. The user can inspect their deleted files in Trash as well as restore them when necessary. There was also some further information regarding the deleted files, such as the date and time of deletion. Sync.com cloud files were kept on at least three distinct servers across two different data centres (Toronto, ON, and Scarborough, ON, Canada). This was to guarantee data redundancy, so that if one server fails, the user may still access their files from another server that is up and running. Furthermore, Sync.com allows you to access your account even if you are not connected to the Internet. All the information and file metadata discovered on Sync.com cloud has a specific function in assisting forensic investigators in acquiring critical evidence for crimes.

### 5.2. Analysing Sync.com Cloud Base Images Created by Different Browsers

To function as a control, basic pictures were prepared before each scenario was developed in this case. This is carried out to guarantee that no associated artefacts or files were present prior to each browser installation and scenario. We conducted keyword searches using various keyword terms to produce this analysis, as indicated in Table 5.

Before each of the installations, the examination revealed that no data and artefacts linked to browser and Sync.com cloud client software files were present.

### 5.3. Analysis of Sync.com Cloud Client Software

According to the prior tests, the Sync.com client software for Microsoft Windows 10 was downloaded from www.Sync.com. The files were then saved in the “C:\Users\[user_name]\Downloads” folder with the name sync-installer. When the program starts, a popup appears, as seen in the image below, instructing the user to install Microsoft.NET 4.5.2 to continue the installation process. An identical thing happened in each of the four virtual machines that were used to install the Sync.com client software. The executable files Sync.com cloud Windows.exe were copied onto the hard disc of the VM established after installing Microsoft.NET 4.5.2.

While the three upload VMs were examined, it was discovered that the Sync.com client software was installed in the “C:\Program Files (x86)” folder. Sync.com sample files and folders were also discovered on the hard drive, in the default Sync.com folder, which was situated at “C:\Users\[user_name]\Sync”. At the same time, as indicated in the diagram below, there was a drive named “Sync” under My Computer.

Using the FTK Toolkit to analyse the files presented above, forensic investigators can select specific files that are required for the inquiry. The examiners can draw conclusions about the time this program was last accessed, generated on the system, or updated based on the study of each timestamp created by those files, such as the date of creation, date modified, and date of last access. Furthermore, the synchronisation of files in the Sync.com Drive with files on online surfing enables investigators to access critical data and evidence for their investigations.

According to McClain, the Dropbox client software includes a file called “filecache.db” that contains a history of synchronised filenames. Furthermore, Google Drive research has revealed that there are files named “sync config.db” and “snapshot.db” that include information and files downloaded and synchronised, as well as details of files related to the cloud storage. The pCloud client program, according to [13], has a file entitled “data.db” that keeps a history of filenames and other related data that are synced with pCloud. As a result, inspection of the Sync.com folder revealed a “cfg.db” file in the “C:\Users\[user_name]AppData\Local\Sync.Config\1.1” folder. As shown in the picture below, the file “cfg.db” and other database files contain a variety of information, including the local path of Sync.com cloud, web surfing information used to access Sync.com cloud, and the number of ports. Examiners can acquire access to the content saved in a certain Sync.com account without logging in as the user if they know where the sync files are located.

Furthermore, “cfg.db” contains important information about the Sync.com cloud storage, such as the Sync.com client software login and password for the account established. This, in turn, can help the examiner with the generally time-consuming process of gathering evidence and retrieving it. A forensic copy of hard drive pictures retrieved from a confiscated computer hard disc, for example, can be used in associated applications to retrieve and scan data. However, this can have a negative impact on Sync.com customers, since information kept on the hard drive, like usernames and passwords, can be readily abused by hackers who obtain remote access to a victim’s computer. After analysing the Sync.com client software, the following Table 6 summarises the files discovered on VM hard discs.

### 5.4. Directory Listing of VM Hard Drives

A directory listing was made for each of the VMs built with Access Data FTK Imager during this test. All the filenames can be seen using this program. In addition, keyword searches were performed on the forensic image files made by each VM in order to filter out the exact keywords of the files on the hard drive. There were no files connected with the Sync.com cloud sample files or the files saved in the Sync.com account before the installation of the Sync.com client software, according to the examination of the four base VMs generated as the controls for this testing. Before the client software installation, there were no additional uploaded .jpg images, nor were there any .pdf or document files synchronised with the Sync.com account.

There were many additional filenames related to Sync.comin the other VMs produced, particularly in Upload-VMs, Access-VMs, Uninstall-VMs, Download-VMs, Eraser-VMs, and CCleaner-VMs. The files were usually found in User’s Desktop, Download folders, Sync Cloud Drive (P:), and the Sync Cloud folder on the hard drive with the address “C\Users\[user_name]\Sync”. The downloaded installation file “Sync installer” was found in the “C:\Users\[user_name]\Downloads” folder in Upload-VMs. In addition, Access-VMs keep files related to Sync.com cloud in the “cfg.db” specified. The timestamps of “cfg.db” change depending on when the Sync.com client program was last used. There were also traces of files uploaded to the Sync.com cloud that were stored in the Sync.com cloud Drive. This is because files and photos uploaded via online surfing are synced with the Sync.com client software and saved in the Sync Cloud Drive (P:) on the computer’s hard drive. The downloaded files were found at “C:\Users\[user_name]\Downloads” in Download-VMs.

The filenames observed after checking the Sync.com account through a web browser were identical to those seen when accessing the account via client software. This is because the file was synced with the user’s local C:\Users\[user_name]\Documents Sync.com cloud folder as well as the Sync Cloud Drive folder “/Sync”.

Finally, the filenames of deleted files were fully removed from the Sync.com cloud folder in the user’s hard drive, according to the analysis of the Eraser-VMs and CCleaner VMs. However, unless the files were explicitly removed from the folder, the downloaded filenames could still be accessed in “C:\Users\[user_name]\Downloads”. The deleted filenames remain in the recycle bin until the user deletes them from the hard drive’s recycle bin. Even if the files are deleted from the recycle bin, they can be readily recovered with the FTK Imager. All of these clues discovered in all of the files on the computer hard disc will undoubtedly aid the forensics investigator in their data analysis procedure. The directory listing in VM hard drives is shown in Table 7.

### 5.5. Link Files of VMs

The analysis of link files showed that filenames with the extension of .lnk associated with Sync Cloud sample files, such as Test.lnk and Test1.lnk, were located at “C:\Users\[user_name]\AppData\Roaming\Microsoft\Windows\Recent Items”, residing in all Upload-, Download-, Eraser-, and CCleaner-VMs. However, there were no other link files found within the four Access-VMs. This indicates that the link files will only be created after the files are downloaded and opened. Table 8 shows the summary of link files residing in VM hard drives.

### 5.6. Prefetch Files Created on VMs After Each Running Application

Forensic investigators can use prefetch files to examine the apps that are executing on a system. When a program is executed for the first time from a location, Microsoft Windows creates a prefetch file to speed up the loading time of the application. As a result, these files include valuable information on the user’s program history.

**Figure 3:**
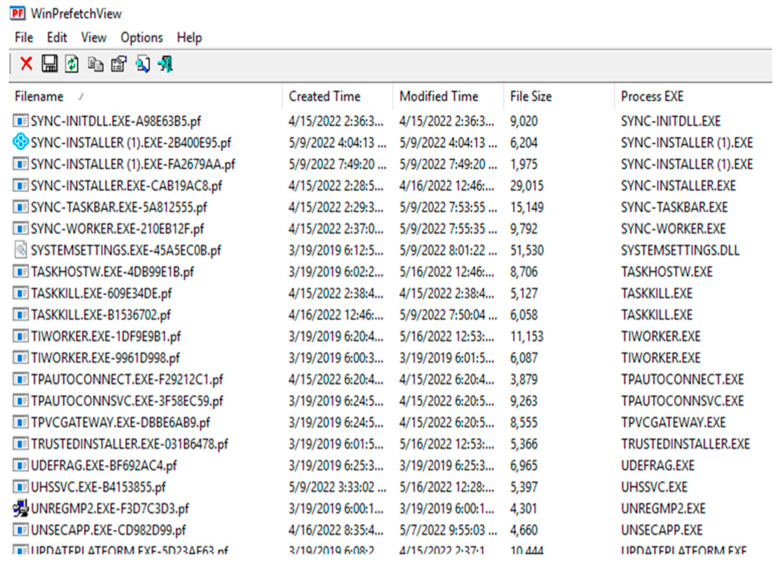
Example of the lists of prefetch files found.

It was discovered, for example, that CCleaner was used before to erase data and uninstall the Sync.com client program from the hard drive. Furthermore, even after the client program was removed, prefetch files remained on the machine. “C:\Windows\Prefetch” was discovered to include prefetch files. WinPrefetchView v1.37 was used to see the prefetch files discovered, as illustrated in Figure 3.

### 5.7. Analysis of Event Logs Files

The built-in Microsoft Windows event viewer was used to examine and analyse the event logs in this study. Throughout the testing period, all of the files in the event viewer were viewed for all of the events that occurred in the VMs. When the Sync.com client software was installed, it was observed that a rule was added to the Microsoft Windows Firewall exception list. Another rule called “Windows Communication Foundation Net.TCP Listener Adapter (TCP-In)” was added to the Microsoft Windows Firewall exception list after installing Microsoft.NET, which was necessary for the installation of the Sync.com client software. Each software installation procedure was discovered to be stored in the event log files “Microsoft-Windows-Windows Firewall with Advanced Security % 4Firewall.evtx” and “Security.tvtx” contained in the “percent SystemRoot percent system32winevtlogs” folder C:Windows\System32\winevtx\logs.

### 5.8. Random Access Memory (RAM) Analysis

FTK was used to acquire VMEM files from memory grabs in each scenario before the VMs were shut down in this study. The word “Sync” was found in the VMEM files collected in all situations, except the Base-VMs, according to the examination of each VMEM file gathered from each scenario. Except for the Base-VMs, the website URL (www.Sync.com) was in all other VMEM files.

Sync.com account information, including the login, was also discovered in Upload-VMs, Access-VMs, and Download-VMs. “>=r....[username]”, “5username[username]”, and “&username = [username]” are all examples of usernames that were discovered. Furthermore, the Sync.com account password was plainly shown in plain text around the words “&password = [password]” and “*eà [password]”. This material can be used as a guide for forensic investigators who are carrying out a keyword search to uncover the criminal’s prospective Sync.com account information. Data carving was also recovered in the thumbnail photographs, as well as incomplete picture files recovered from the Sync.com cloud sample files in the VMEM files for Access-, Upload-, and Download-VMs.

### 5.9. Thumbcache Files

Thumbcache files are databases that contain thumbnail pictures of your system’s varied content. When the mouse is held over an image in a folder, for example, a thumbnail preview of that picture is generated. According to the thumbcache files for the four base VMs, no Sync.com sample photographs existed before the installation and use of the Sync.com client software. Aside from that, the Sync.com sample photographs stored in Access-VMs and CCleaner-VMs were not detected. Other VMs, such as Upload-VM, Download-VM, and Eraser-VMs, did, however, contain examples of thumbnails for the stored Sync.com sample files. This indicates that thumbnail pictures are only saved in thumbcache files after certain files have been downloaded, browsed, or posted into the account.

### 5.10. Network PCAP Files

Throughout the live network capture during each use of Sync.com cloud using multiple browsers, PCAP files or files, also known as Packet Capture data, were produced using Wireshark and Network Miner 2.0 as part of this research. PCAP files were intended to aid forensic investigators in analysing data networks and packet sniffing characteristics [23]. The examiner can extract information about network activity as well as file remains linked with Sync.com cloud operations by analysing network capture files. The network activity was watched via port 443, which was https. When utilising the client software or a web browser to access a Sync.com account, research indicates that a session with an IP range of 192.40.81.0–192.40.81.255 was registered under Sync.com from Canada. Then, on Port 443, another session with an IP range of 54.146.35.204 to 54.146.35.255 was registered under sync.com from the United States.

### 5.11. Anti-Forensic Technique and the Uninstallation of Sync.com Cloud Client Software

CCleaner and Eraser were both used to erase the downloaded files from the Sync.com account, as well as uninstall the Sync.com client software as part of the testing. Even though the client software had been uninstalled, several data remnants associated with Sync.com cloud were still found on the hard drives, including Sync.com files, Sync.com cache files, web-browsing history, and so on. This suggests that anti-forensic tools such as CCleaner and Eraser were unable to entirely remove all traces of files related to Sync.com cloud. This can help forensic investigators obtain evidence from user hard discs.

As a result of the data and file remnant analysis, only the files installed in the “C:\Program Files (x86)” folder, as well as Sync.com sample files and folders, were found on the hard drive. However, the Sync.com folder was erased from “C:\Users\[user_name]\Sync”. The Sync.com folder could still be accessible in the computer hard drive at “C\:Users\[user_name]\Sync”. Furthermore, the “C:\Users\[user_name]\AppData\Local\Sync.Config\1.1” folder included all of the client software caches, as well as “cfg.db” and “State.db.backup”, which contained the most critical information about the account information. This demonstrates that various files vestiges remained unaltered by the removal of the Sync.com client program. The files found in VM hard drives following the uninstallation of the Sync.com client program and the files connected with it are summarised in Table 9.

### 5.12. Presentation of Evidence

After users viewed, downloaded, or saved items in the Sync.com account, a variety of data remains were discovered on the hard discs of the VMs in this study. All of the evidence acquired was enough to lead forensic investigators through their case investigation. This is because the forensic investigator was able to locate the file location that included all of the evidence required for the inquiry. Knowing the location of the evidence allows the forensics investigator to go right to the site of retrieving the evidence, reducing the time it takes to solve a case.

Thus, information such as Sync.com users, passwords, session IDs, network traffic, prefetch file listings, link files, and browser history were shown to be definite clues to identify the content of the files that may be significant in solving a particular case. In addition, volatile data analysis proved to be effective and decisive in detecting Sync.com account access as well as the networking equipment utilised to join the Sync.com account. This is because when clients visit the account via browsers, the devices used to access and synchronise with the Sync.com account are shown. This is significant since each device synced with the Sync.com account may contain evidence related to the cases under investigation. The results of the analysis are summarised in Table 10 below.

## 6. Comparative Analysis

Comparing our forensic approach with existing methods revealed that our methodology excelled in its comprehensive ability to recover a wide range of digital artefacts, setting it apart from previous studies. The use of FTK Imager enabled us to retrieve a complete suite of digital traces, including cache files, usernames, passwords, database files, log files, cookies, and deleted files—addressing the gaps often seen in other configurations. Remarkably, even with the use of data deletion tools such as CCleaner and Eraser to remove remnants, FTK Imager demonstrated its powerful capability to recover deleted data effectively.

For example, studies using Amazon Cloud Drive and Google Drive ([15,16]) leveraged tools such as EnCase and BleachBit, but they showed limitations in recovering certain types of data, such as cookies or deleted files. Our method overcame these limitations, showcasing a complete retrieval of all data types tested. Similarly, Dropbox configurations on Linux ([17]) lacked recovery capabilities in critical areas, such as passwords and database files, while our approach successfully addressed these gaps. The experiment using Microsoft OneDrive and Amazon Cloud Drive ([18]) provided better results but lacked the flexibility and thoroughness that our configuration achieved with our tool selection.

By integrating FTK Imager alongside Eraser and CCleaner, we not only matched but exceeded the capabilities demonstrated in other studies, providing a seamless recovery of all tested digital artefacts. Our approach’s robustness was especially apparent when compared to other tools such as OS Forensics, Internet Evidence Finder, and Redline ([19]), which, while effective, do not consistently recover all artefacts across various cloud platforms. Even more recent studies using FTK Imager on pCloud ([22]) showed partial results, especially in the areas of database and log file recovery, while our methodology covered these elements comprehensively.

The strength of our experiment lies not only in its completeness but also in its practical relevance. By ensuring the recovery of all tested artefact types, our approach offers a reliable framework that digital forensic investigators can depend on for thorough, accurate analyses across popular cloud platforms. This comprehensiveness makes our experiment a valuable resource in digital forensics, emphasising data integrity and minimising data loss in investigative scenarios. Table 11 summarises the comparison between our study and the most relevant works in terms of cloud types, operating systems, browsers, forensic tools, and experimental findings.

## 7. Conclusions and Future Work

Cloud computing and cloud storage services have grown in both acceptability and popularity as the technology era progresses because they are convenient for individuals all over the world. However, cloud technology is making it more difficult for forensic investigators to cope with cybercrime data. This is because data may be uploaded and accessed from several devices without leaving a trail. As a result, determining the sorts of cloud service providers utilised by criminals, as well as the user data required to help the investigation, is critical. This will aid forensic investigators in locating critical data, as well as extracting and preserving it in a forensically sound manner.

Following the completion of all scenario testing, it was determined that this research satisfied all the objectives outlined. The first aim of this study, “Retrieving and Identifying Remnants of Artefacts on Local Devices Using Sync.com cloud”, was met, which was to investigate existing digital evidence-collecting methodologies, as well as the location and data fragments left on a user’s computer prior to using a cloud storage service. Literature reviews were analysed in this study to examine and obtain information about previous relevant research. Each article’s research findings, techniques, limitations, and conclusions were evaluated and analysed for suggestions and direction in conducting this study. Aside from that, the general qualities of the leading cloud service providers were established to extend our perspective on current cloud technology.

Furthermore, the second goal of this study, which was to suggest a proactive strategy for locating and removing data fragments left on a user’s computer prior to using a cloud storage application for fresh cloud storage, was satisfied. We were able to identify many methods for gathering relevant information because of our research, which may be useful to forensic investigators in solving cases. In addition, the third aim of this study, which was to test and develop a technique for locating and implementing data fragments left on a user’s computer prior to using a cloud storage application for fresh cloud storage, was satisfied. We discovered that when web access was carried out using several browsers, the possible data residue on a Microsoft Windows 10 PC may be obtained from the browser history utilising Sync.com cloud as a case study. Client software files, prefetching files, link files, network traffic capture, and memory captures were effectively detected based on the usage of Sync.com Cloud for each activity carried out by a user, as previously stated.

When we investigated the use of Sync.com cloud storage, the initial stages included the identification of a cloud service and user account. This may enable investigators to identify the location of data. In this research, we found that an investigator can identify Sync.com cloud Drive account use by undertaking keyword searches and examining the test file’s locations to locate relevant information. The remnants of cloud activity can be found on local machines. It could be valuable for the forensic examiners. We found the remnants in local folders. The username, the cache files, and log activity helped in recovering the deleted files and data. We identified the locations of data and files to determine user details and cloud storage information related to the use of the Sync.com Cloud Drive in our research.

This research was limited to the case study of Sync.com cloud only. More research needs to be carried out in the future to examine other newly developed cloud storage services that are new in the market by employing the same methodology in future studies. This is to test the suitability of this methodology when applied to other cloud service providers.

In addition, there are plans for further research to broaden the research area to include more experiments regarding mobile devices and tablets that are synchronised with cloud services accounts. Furthermore, future research opportunities also include carrying out testing and experimenting with the latest operating systems, which include Microsoft Windows 11.

## Figures and Tables

**Table 1:** Summary of each cloud’s storage services.

	Dropbox	Google Drive	Microsoft OneDrive	pCloud	Sync.com Cloud
Size of free storage	2 GB	15 GB	5 GB	10 GB	5 GB
Security	Client encrypted files, Geo-Redundant storage and folder	Data stored on Drive is, similar to Apple, encrypted in 128-bit AES	Encryption at rest is available on OneDrive, but only for business users	pCloud uses TLS/SSL encryption, applied when information is transferred	Data secured using zero-knowledge encryption with AES 256-bit file encryption and two-factor authentication
File Versioning	Yes	Yes	Yes	Yes	Yes
Area of specialisation	Compatibility with other services	Data syncing between devices	Offering real-time collaboration features for Microsoft Office 365 Included when purchasing 1 TB.	With pCloud, your valuable files are accessible even offline	You can access to your file anytime from anywhere, your file can be supported with a password; you can also upload many files at the same time and they are accessible offline
File size restriction	10 GB with websites only	5 TB	10 GB	Unlimited	There is no file size restriction.
OS supported	Microsoft Windows, Mac, Linux, Android, iOS, Microsoft Windows Phone, BlackBerry, Kindle Fire	Microsoft Windows, Mac, Android, iOS	Microsoft Windows, Mac, Android, iOS, Windows Phone	Microsoft Windows, Mac, Android, iOS, Linux	Microsoft Windows, MacOS, mobile apps for Android and iOS.

**Table 2:** Software Requirements.

Software Required	Description
VMWare Player, v16.2.0	For testing purposes, a virtualized desktop is used to run an operating system.
Multiple browsers including Microsoft Edge (ME), Google Chrome (GC), and Mozilla Firefox (FF)	To create and access Sync.com cloud application account in a range of ways, including different browsers.
Ccleaner, v5.81.8895	To clean specific files.
Eraser, v6.2.0.2992	To erase specific files.
Sync.com cloud application v4	To gain access to the information saved in the cloud application.
FTK Imager, v 7.5.1	E01 files for each VM will be created using this tool.
Encase and FTK v 7.5.1	Use to analysing the images from VM.
Wireshark	To capture the PCAP files.
Process Monitor	Use to keep track of changes to files and the registry.
Window 10 Pro 64 bit	Platform for research study development.
Microsoft Word	Drafts of proposal and to write document.

**Table 3:** Hardware Requirements.

Specifications	Description
Laptops (ASUS K43SD, Intel Core i5 2450 M 2.5 GHz, 6 GB DDR3 SDRAM, 2.5” SATA 500 GB 5400 rpm)	Used for the whole research study that included documentation and result comparison.
External Hard Disk of Toshiba 500 GB	To back-up study files.
16 GB Kingston thumb drive	To transfer files.

**Table 4:** Types of VMs needed.

Virtual Machines	Details
Base-VM, which includes ME, MF, and GC	2 GB RAM, 20 GB Hard Disk Drive, Microsoft Windows 10 Home Basic SP1. Microsoft Edge (ME), Mozilla Firefox (FF), and Google Chrome are the browsers that are installed for each test (GC).
Upload-VM ME, GC, and FF	Download and install the Sync.com Cloud Microsoft Windows Client program. The test account was accessed. Data from Enron were uploaded to a user’s Sync.com cloud account.
Uninstall-VM ME, FF, and GC	Uninstall the Sync.com cloud client program utilising the Upload-VM via the Microsoft Windows Start Menu option.
Access-VM ME, GC, and FF	Browser used to sign in to the user test account on the Sync.com website at www.sync.com. Each file in the Sync.com cloud account storage was accessed but not downloaded on purpose.
Download-VM ME, GC, and FF	Browser used to sign in to the user test account on the Sync.com website at www.sync.com. Each file was downloaded and opened on the VM Hard Drive Desktop.
Eraser-VM ME, GC, and FF	The Sync.com cloud and Enron data were deleted using Eraser software on each copy of the Download-VMs.
CCleaner-VM ME, GC, and FF	Each copy of the Eraser-VMs had CCleaner downloaded, installed, and run with default parameters.

**Table 5:** List of keyword search terms.

Keyword Categories	Keyword
Browser	Google Chrome, Firefox, Microsoft Edge
Software	Sync Cloud, Sync.com
Pictures uploaded	Test.png, Test1.png
PDF uploaded	Test.pdf
Documents	Test.doc, Test.pptx, Test.txt
Password	123456789test, test, 123456789

**Table 6:** Summary of files found residing in VM hard drives after the analysis of Sync.com cloud client software.

No.	Files Name	File Location	Description
1	Sync installer .exe	“C:\Users\[user_name]\Downloads\” folder	Sync.com client software installation files.
2	Sync.com cloud client software installed	“C:\Program Files (x86)\” folder	Sync.com client software installed
3	“cfg.db” “cfg.db.lock” “state.db” “states.db” “state.db.backup” “State.db.changecount” “State.db-shm” “statedb-wal” “states.db.lock” “filecache.db” “sync config.db” “snapshot.db”	“C:\Users\[user_name]\AppData\Local\Sync.Config/1.1”	Local path of Sync.com, web browsing information used to access Sync.com cloud with the number of ports, username, files stored, metadata of the files stored on the cloud itself.

**Table 7:** Summary of directory listing in VM hard drives.

No	VMs	File Names	Location of the Files
1	Upload-VMs	Sync installer	C:\Users\[user_name]\Downloads\
2	Access-VMs	cfg.db, data.db.backup synced files Synced files	“C:\Users\[user_name]\AppData\Local\Sync.Config\1.1” “C:\Users\[user_name]\AppData\Local\Sync.Logs” C:\Users\[user_name]\Documents\ Sync
3	Download- VMS	Downloaded files	Sync Cloud Drive (P:\) C:\Users\[user_name]\Downloads\
4	CCleaner-VMs	Deleted files (Test1.png, Test1.png, Test.pdf, Test.pptx, Test.doc, Test.txt)	C:\Users\[user_name]\Downloads\
5	Eraser-VMs	Deleted Files (Test1.png, Test1.png, Test.pdf, Test.pptx, Test.doc, Test.txt)	C:\Users\[user_name]\Downloads\

**Table 8:** Summary of link files residing in VM hard drives.

No	Files Names	File Location
1	(Test.png, Test1.png, Test.pdf, Test.pptx, Test.doc, Test.txt)	“C:\Users\[user_name]\AppData\Roaming\Microsoft\Windows\Recent”

**Table 9:** Summary of files found in VM hard drives after the uninstallation of Sync.com Cloud client software and the files associated with it.

No	Name of Files	Location of Files
1	Sync Cloud sample files deleted	C:\Program Files (x86)\
2	Sync Cloud folder	C:\Users\[user_name]\Documents\Sync
3	cfg.db State.db.backup	C:\Users\[user_name]\AppData\Local\Sync.

**Table 10:** Summary of the analysis findings.

Type of VMs	Data Remnant Found				
	Password	Username	Software	Sample Files	Keyword Search Term
Base VMs	Nil	Nil	Nil	Nil	Nil
Upload-VMs	Found in RAM	Found in RAM	Sync installer.exe was found after the download. The location of client software installation and the Sync.com sample files uploaded were found.	Files were found in prefetch files, link files, and so on.	Multiple matches of keyword search obtained.
Access-VMs	Found in RAM	Found in RAM	Nil	The information of Sync.com cloud software accessed was found residing in cookies, the browsing history, page files, and unallocated spaces.	Multiple matches of keyword search obtained.
Download -VMs	Found in RAM	Found in RAM	Nil	The downloaded files were stored in the hard drives of VMs	Multiple matches of keyword search obtained.
Eraser-VMs	Nil	Nil	Nil	The information of Sync.com software accessed was found residing in cookies, the browsing history, page files, and unallocated spaces. The deleted files were still able to be found in unallocated spaces.	Multiple matches of keyword search obtained.
CCleaner- VMs	Nil	Nil	Nil	The information of Sync.com software accessed was found residing in cookies, the browsing history, page files, and unallocated spaces. The deleted files were still able to be found in unallocated spaces.	Multiple matches of keyword search obtained.

**Table 11:** Shows a comparison of the analysis findings between our study and others.

Reference	Cloud	OS	Forensic Tool	Findings						
				Cash Files	Username	Password	Database File	Log Files	Cookies	Deleted Files
[15]	Amazon Cloud Drive	Microsoft Windows 10	EnCase BleachBit	Yes	Yes	Yes	Yes	Yes	No	Yes
[16]	Google Drive	Microsoft Windows XP, 7, 8	EnCase CCleaner	Yes	Yes	Yes	Yes	Yes	Yes	Yes
[17]	Dropbox	Linux -Ubuntu 16.04 LTS	Testdisk PhotoRec	No	Yes	No	Yes	Yes	No	Yes
[18]	Microsoft One Drive and Amazon Cloud Drive	Microsoft Windows 7	No	Yes	Yes	Yes	Yes	Yes	Yes	No
[19]	Mega drive and IDrive	Microsoft Windows 7	OS forensics, Internet Evidence Finder & Redline	Yes	Yes	Yes	Yes	Yes	Yes	No
[22]	pCloud	Microsoft Windows 7	FTK imager Eraser and CCleaner	Yes	Yes	Yes	Yes	Yes	Yes	Yes
Ours	Sync.com Cloud	Microsoft Windows 10	FTK imager Eraser and CCleaner	Yes	Yes	Yes	Yes	Yes	Yes	Yes

## Data Availability

Data are contained within the article.
